# Infertility Improvement after Medical Weight Loss in Women and Men: A Review of the Literature

**DOI:** 10.3390/ijms25031909

**Published:** 2024-02-05

**Authors:** Polina Pavli, Olga Triantafyllidou, Efthymios Kapantais, Nikolaos F. Vlahos, Georgios Valsamakis

**Affiliations:** 1 2nd Department of Obstetrics and Gynecology, National and Kapodistrian University of Athens, “Aretaieion” University Hospital, 11528 Athens, Greece; p92pavli@gmail.com (P.P.); triantafyllidouolga@gmail.com (O.T.); gedvalsamakis@yahoo.com (G.V.); 2 Department of Diabetes and Obesity, Metropolitan Hospital, 18547 Athens, Greece; ek@kapantais.gr

**Keywords:** medically induced weight loss, fertility improvement, GLP-1 RA, obesity and fertility outcomes

## Abstract

Infertility is a modern health problem. Obesity is another expanding health issue associated with chronic diseases among which infertility is also included. This review will focus on the effects of weight loss by medical therapy on fertility regarding reproductive hormonal profile, ovulation rates, time to pregnancy, implantation rates, pregnancy rates, normal embryo development, and live birth rates. We comprised medicine already used for weight loss, such as orlistat and metformin, and emerging medical treatments, such as Glucagon-Like Peptide-1 receptor agonists (GLP-1 RA). Their use is not recommended during a planned pregnancy, and they should be discontinued in such cases. The main outcomes of this literature review are the following: modest weight loss after medication and the duration of the treatment are important factors for fertility improvement. The fecundity outcomes upon which medical-induced weight loss provides significant results are the female reproductive hormonal profile, menstrual cyclicity, ovulation and conception rates, and pregnancy rates. Regarding the male reproductive system, the fertility outcomes that feature significant alterations after medically induced weight loss are as follows: the male reproductive hormonal profile, sperm motility, movement and morphology, weight of reproductive organs, and sexual function. The newer promising GLP-1 RAs show expectations regarding fertility improvement, as they have evidenced encouraging effects on improving ovulation rates and regulating the menstrual cycle. However, more human studies are needed to confirm this. Future research should aim to provide answers about whether medical weight loss therapies affect fertility indirectly through weight loss or by a possible direct action on the reproductive system.

## 1. Introduction

Infertility is a health problem that has occupied the medical community in numerous ways, and it is estimated by the World Health Organization (WHO) to affect around 17.5% of the adult world population in 2023 and approximately 1 out of 7 couples in the UK and the USA [1]. Infertility is defined as failure to achieve a clinical pregnancy after 12 months or more of regular unprotected sexual intercourse [2]. The fertility outcomes constitute a key point and can be evaluated by multiple final endpoints: the reproductive hormonal profile, ovulation rates, time to pregnancy (TTP), conception and implantation rates, pregnancy rates, normal embryo development, and live birth rates (LBRs).

Obesity is another constantly expanding health issue of the modern era. The World Health Organization has defined it as an “abnormal or excessive fat accumulation that may impair health”, which results from an energy imbalance between the consumption and spending of calories [3]. Its prevalence has tripled worldwide over the past 30 years. The term “overweight” is defined as an increased body mass index (BMI) over 25 kg/m^2^ and the term “obesity” is defined as a BMI over 30 kg/m^2^ [4]. Obesity is associated with several chronic diseases, among which infertility is also encompassed [5].

The primary infertility outcomes concerning obesity are anovulation, abnormal reproductive hormone levels, impaired endometrial decidualization, 4–5% decreased pregnancy rates [6], and longer times to achieve clinical pregnancy [7], with this delay getting significant when the BMI is over 35 kg/m^2^ [8]. Moreover, because of obesity, in vitro fertilization (IVF) outcomes become poorer [9], with lower implantation rates [10,11], increased risk of miscarriage [12,13], and higher doses of ovulation induction medications [14], with the need for a longer stimulation time [15].

Currently, the overwhelming percentage of literature remains controversial regarding the field of medical weight loss and infertility. Some studies outline a positive effect of weight improvement on fertility, as weight reduction seems to increase the chances of conception, regardless of menstrual regularity, parity, smoking, age [16], and race [8]. However, other studies present no significant data [17]. Regarding medical therapeutic choices, there are older drugs that have been used for weight loss over the past years, such as orlistat and metformin, and newer medicines, such as Glucagon-Like Peptide-1 receptor agonists (GLP-1 RA), which have only recently been used for weight improvement. Nevertheless, there are not enough research data about the effects of those medical therapies on the fertility of women and men. In addition, it is verified that all of the existing medical therapies should not be used during the period of known or planned pregnancy and should be discontinued in such a case [18]. Furthermore, there is not a clear recommendation about how much weight should be lost in cases of infertile, morbidly obese women, during which period of time, how long before the onset of conception, and through which specific medical treatment.

In this review, we clarify whether medical weight loss is efficient regarding fertility improvement, upon which fertility outcomes, which medical treatment is the most effective one, how much weight should be lost and during which specific period of time before trying to attain a successful clinical pregnancy.

## 2. Pathophysiology: Obesity Mechanisms Leading to Infertility

There are a few potential mechanisms that interpret the pathophysiological phenomenon of obesity, leading to reduced fecundity. Firstly, pathological BMI has been associated with anovulation due to dysregulation of the hypothalamic–pituitary–ovarian (HPO) axis [19,20] and decreased amplitude of luteinizing hormone (LH) pulsatility [21]. Insulin resistance (IR) and hyperinsulinemia of obese women, reduced sex-hormone-binding globulin (SHBG) plasma concentrations, increased circulating free androgens, and estrogen conclude in the outcome of suppressed follicle-stimulating hormone (FSH) release, impeding normal follicular induction and ovulation [19,20]. Abnormally affected concentrations of leptin, an adipokine produced in the adipose tissue, affect steroidogenic pathways in granulosa cells and inhibit folliculogenesis in a dose-dependent manner [19,22,23,24].

Research data from the bibliography suggest that lipotoxicity and an already coexisting low-grade inflammatory state in obese women affect important reproductive events, such as the time of ovulation and the invasion of the trophoblast [25,26]. Additionally, morbidly obese women present a lower oocyte yield [27], as there is a molecular, altered mitochondrial function in the existing oocyte and an endoplasmic reticulum stress, which leads to increased granulosa cell apoptosis [28]. The pathophysiological mechanisms of lipotoxicity and notably elevated leptin concentrations of obese women may also exert a direct negative effect on the developing embryo [29,30]. There are also data regarding impaired endometrial decidualization due to obesity [31]. Moreover, chronic dysregulation of leptin pathways may negatively affect implantation and modulate endometrial receptivity in obese women [32].

Regarding male infertility, the proposed pathophysiological mechanisms include a reduction in sperm concentration, morphology and progressive motility [33,34], fewer sexual encounters [35], erectile dysfunction (ED) [36], and increased testicular temperature [33]. A deleterious effect on spermatogenesis [37,38] and on the normal function of the HPO axis [34] seems to be as a result of increased estrogen plasma concentrations and reduced testosterone plasma concentrations after peripheral aromatization [39].

## 3. Weight Loss Interventions and Female Fertility

### 3.1. Weight Loss through Medical Therapy on Female Fertility

Pharmacotherapies that aim primarily at weight improvement include several medicines that have been included over the years in the treatment of obesity. Orlistat; phentermine and topiramate combination; sibutramine; metformin; and GLP-1 RA, such as exenatide, dulaglutide, liraglutide, semaglutide, tirzepatide, and a combination of GLP-1 RA with amylin analogs, are within the range of these therapeutic choices. However, it is still debatable whether these medicines may be determining factors for weight loss related to improved fecundity. Currently, the general recommendation for those medications is to be discontinued when a spontaneous pregnancy is succeeded. In this review, we provide all the ongoing data in relation to each medical therapy used in the field.

#### 3.1.1. Orlistat

Orlistat is an inhibitor of gastrointestinal lipase and has been approved for the long-term management of obesity since 1999 [40]. It is used orally at dosages of 60 mg or 120 mg per day, but side effects, such as gastrointestinal effects, have reduced its usage [40]. A recent prospective study, which involved 120 obese, infertile women, 21–35 years old with BMI over 25 kg/m^2^, aimed to evaluate the effects of orlistat on reproductive hormonal profile and pregnancy rates. Participants were divided into two groups: the first group underwent orlistat therapy of 120 mg twice per day for 6 months and the second group was recommended to follow lifestyle modifications (diet, exercise, and behavioral measures). The results highlighted a statistically significant increase in post-treatment LH plasma concentrations in the orlistat group. In addition, there was a statistically significant reduction in pre-and post-treatment free testosterone serum concentrations and anti-Müllerian hormone (AMH) concentrations in the orlistat group. Moreover, there was a significant difference between the two groups regarding pregnancy rates, with the orlistat group presenting better outcomes. As far as weight change is concerned, a statistically significant difference between pre- and post-treatment weight was observed in both groups [41]. In another study with the administration of orlistat in women diagnosed with polycystic ovary syndrome (PCOS), a beneficial effect of orlistat therapy for weight loss and the female reproductive profile was observed. Sixty-one obese women with PCOS and twenty overweight and obese controls were included in the study [42]. The participants followed an energy-restricted diet, physical exercise plus the administration of 120 mg of orlistat, 3 times per day, for 24 weeks. As far as the hormonal profile of PCOS women is concerned, the concentrations of serum LH and SHBG were significantly increased, while the testosterone plasma concentrations were significantly reduced. In addition, orlistat administration combined with lifestyle modifications resulted in remarkable weight loss, and the BMI was significantly lower in both the PCOS and control women, especially during the first trimester follow up (from 34.83 ± 6.39 to 31.90 ± 6.09 kg/m^2^) [42]. An older prospective clinical study included 18, ideal for age and BMI, women with a PCOS diagnosis and 14 obese controls, with normal menstrual cycles [43]. The recommendation was for all participants to follow an energy-restricted diet plus medical therapy with 120 mg of orlistat, 3 times per day, for 24 weeks. The results suggest that the testosterone plasma concentrations were statistically significantly reduced in the PCOS group during the first trimester of the study, from 83.26 ± 6.86 ng/dL to 61.50 ± 4.97 ng/dL. Furthermore, orlistat administration plus diet for 24 weeks resulted in significant weight improvement, with the BMI in the PCOS group improving from 36.00 ± 1.29 kg/m^2^ to 30.36 ± 1.18 kg/m^2^ [43].

Regarding pregnancy rates, in a randomized control trial (RCT) of 90 PCOS women, the administration of orlistat resulted in positive effects when compared with the controls. The participants were randomized equally to either orlistat or metformin therapy, combined with lifestyle interventions, compared with the controls, who followed only the lifestyle modification program. The medical interventions were as follows: 500 mg metformin three times per day, following a gradual increase, 120 mg orlistat therapy twice per day plus fertility fitness program of diet, exercise, and lifestyle modification for 3 months. In relation to weight improvement, both treatments had similar results as far as total body weight change and BMI were concerned, with a reduction of 7.81 ± 0.66 kg in the orlistat group and 7.78 ± 0.57 kg in the metformin group. Ovulation rates were 33.3% and 23.35% in the orlistat group and the metformin groups respectively, without a statistically significant difference. Conception rates were 40%, 16.7%, and 3.3% in the orlistat, metformin, and control groups, respectively [44].

Another double-blind, placebo-control RCT aimed to highlight whether orlistat-induced weight loss before IVF would improve LBRs among overweight or obese women [45]. A total of 877 infertile, obese women were included and randomly treated either with orlistat or placebo for 4 to 12 weeks. The results evidenced that the LBRs were not significantly different between the groups, whereas there were also no significant differences in relation to pregnancy rates or clinical pregnancies. There was a decrease in total body weight of 2.49 kg in the orlistat group and 1.22 kg in the placebo group, with a statistically significant difference to be mentioned [45].

#### 3.1.2. Phentermine/Topiramate Combination

Phentermine is a sympathomimetic amine that suppresses hunger and stimulates energy expenditure by inducing catecholamine release in the hypothalamus. The mechanism of action of topiramate, which is an antiepileptic medicine, includes modulation of central voltage-gated ion channels, stimulation of gamma-aminobutyric acid (GABA) activity, and inhibition of glutamate receptors and carbonic anhydrase activity. At present and until greater clinical experience and longer-term outcomes become available through clinical trials, the effects of this medical combination on primary fertility outcomes remain uncertain [46].

#### 3.1.3. Metformin

Metformin is a biguanide antihyperglycemic medication, which has had a proven use in diabetes mellitus (DM) therapy over the years. Regarding weight loss and improved fertility, there are some special data on the bibliography.

A prospective, single-blind, randomized, pilot study compared a 16-week medical therapy of 2000 mg metformin per day to 5 mg saxagliptin daily in 34 women with PCOS and prediabetes. There were three groups of participants: the metformin group, the saxagliptin group, and the combined-therapy group. Body mass index, waist circumference, and insulin sensitivity were improved in all groups, with BMI becoming statistically significantly reduced in the saxagliptin group and the combined group, pre and post-therapy, from 37.2 ± 6.8 to 36.7 ± 7.4 kg/m^2^ and from 43.8 ± 10.5 to 42 ± 10.2 kg/m^2^, respectively. Moreover, the menstrual cyclicity was more regular in the combined therapy group, while dehydroepiandrosterone-sulfate (DHEAS) and testosterone plasma concentrations and free androgen index (FAI) were significantly lower after the 16-week treatment. Furthermore, SHBG plasma concentrations were increased, but without a significant difference and not in the metformin group [47].

#### 3.1.4. Exenatide

Exenatide is the first GLP-1 RA; it was approved in 2005 to treat DM type 2 and was later used for the treatment of obesity [48]. It is prescribed as a subcutaneous injection, at a dosage of 5 micrograms or 10 micrograms twice daily. It was produced incidentally by the peptide exendin-4 when it was isolated by the saliva of a venomous lizard (*Heloderma suspectum*). Exendin-4 is homologous to GLP-1, so it is also able to bind to GLP-1 receptors. It acts by increasing insulin secretion, suppressing glucagon hypersecretion, and reducing food intake, by delaying gastric emptying [49]. Exenatide affects the female reproductive system by ameliorating the hormonal profile: it increases FSH and SHBG plasma concentrations, reduces testosterone serum concentrations, and it improves ovulation rates and menstrual cyclicity in women with PCOS [50]. Regarding diabetic patients, exenatide provides positive effects on the male reproductive system, by increasing testosterone plasma concentrations and ameliorating sexual function [51].

Sixty overweight/obese women diagnosed with PCOS were randomly allocated to receive either metformin or exenatide or a combined medical therapy for 24 weeks, in a prospective study context. There were three intervention groups, which followed treatment with 1000 mg metformin twice daily, 10 μg exenatide twice daily, or 1000 mg metformin twice per day plus 10 μg exenatide twice per day as a combined therapy. Firstly, weight betterment was statistically significant among the three groups, with the best results presented in the combined therapy group. A mean weight loss of 6 ± 0.5 kg was presented in the combined therapy group, 3.2 ± 0.1 kg in the exenatide group, and 1.6 ± 0.2 kg in the metformin group. Regarding hormonal profile, the total testosterone plasma concentrations and FAI were significantly decreased among the three therapy groups, whereas the SHBG serum concentrations improved, without a statistically significant difference. In addition, menstrual cyclicity improved significantly in the combined treatment group [52].

Another 24-week open-label RCT evaluated 176 overweight/obese PCOS women, who were randomly allocated to receive either 1000 mg twice per day or 10 μg exenatide twice per day for the initial 12 weeks. A second period of 12 more weeks of medical therapy followed, during which all women followed metformin-only therapy. During the first half of the period, the exenatide group showed statistically significant weight improvement compared with the metformin group: a total body weight reduction of 4.29 ± 1.29 kg versus 2.28 ± 0.55, respectively, and a total fat reduction of 4.67 ± 0.09% versus 1.11 ± 0.32%, respectively, were mentioned between the groups. The menstrual cyclicity was improved in the exenatide group in comparison with the metformin group. During the last half of the period, there was a higher rate of spontaneous pregnancy mentioned in the group that received exenatide therapy first followed by metformin therapy only during the second part of the study [53]. A similar RCT included 160 obese or overweight, infertile PCOS women, who were randomized into two groups: a group of 5 μg of exenatide therapy twice daily, which was increased to 10 μg twice daily after 4 weeks, and a group of therapy of 500 mg metformin twice per day, which was gradually multiplied up to 1000 mg twice daily. These therapeutic choices lasted 12 weeks. In the following 12 weeks, all participants were treated with metformin-only therapy of a similar starting dose of 500 mg twice daily, which was changed to 1000 mg twice daily gradually. Participants, who were randomized in the metformin group from the beginning of the study, continued their first therapeutic dose, while at the same time, they were encouraged to follow lifestyle interventions. The primary outcome was spontaneous pregnancy rates. The results outlined that the total pregnancy rates were 79.2% in the exenatide group and 76% in the metformin group, without significant differences. Regarding weight improvement, 12 weeks after the beginning of the study, there was a statistically significant difference of weight loss and BMI improvement between the groups. Exenatide therapy presented better results: the weight loss was 5.21 ± 3.94 kg in the exenatide group in contrast with a weight reduction of 3.55 ± 2.13 kg in the metformin group and improvement of BMI in each group was 2.16 ± 1.53 kg/m^2^ and 1.39 ± 0.89 kg/m^2^, respectively [54].

As far as PCOS obese women are concerned, one case report study has also been published, trying to evaluate the effects of medical therapy on fertility in relation to pregnancy rates and LBRs. In 2016, one obese, infertile woman with PCOS underwent medical therapy with 20 μg of exenatide daily for two months, after which ovulation induction was successfully followed by an ongoing singleton pregnancy with a fetal heartbeat, confirmed by transvaginal ultrasound [55].

#### 3.1.5. Dulaglutide

Dulaglutide was initially approved by the Food and Drug Administration (FDA) in 2014 for the treatment of DM type 2. In 2020, it was also approved for use in patients with multiple cardiovascular risk factors. It is a once-weekly, subcutaneous GLP-1 RA of 0.75 mg or 1.5 mg. In 2020, the FDA approved two more doses of the medication, 3.0 mg and 4.5 mg, demonstrating weight benefits [56]. Dulaglutide was designed using recombinant DNA technology, as a polypeptide analog of GLP-1 (7–37) that is covalently linked to each Fc-arm of human immunoglobulin G4. The following substitutions were made on the GLP-1 (7–37) chain: Ala was replaced by Gly at position 8, Glu by Gly at position 22 and Arg by Gly at position 36 [57]. A recent animal study suggested that dulaglutide improves hyperandrogonemia and ovarian function in medically induced PCOS rats when compared with the controls, in a dose-dependent manner [58]. In this animal model, there were four therapy groups, depending on the dulaglutide dosages, which were provided once per week subcutaneously for 3 weeks: the PCOS placebo group, the PCOS on 50 μg/kg of dulaglutide, the PCOS on 150 μg/kg of dulaglutide, and the PCOS on 450 μg/kg of dulaglutide. The intervention group presented a significantly reduced total body weight in a dose-dependent way, while the androgen concentrations of the dulaglutide group were also significantly decreased. Likewise, there was a statistically significant increase in SHBG plasma concentrations in the intervention group. Dulaglutide therapy in medically induced PCOS rats seemed to improve the ovarian function and morphology, as a reduction in the development of small follicles and cystic follicles has been mentioned [58].

#### 3.1.6. Liraglutide

Liraglutide is the second GLP-1 RA, and it was approved in 2009 in Europe and in 2010 in the USA for the treatment of DM type 2 at doses of 0.6, 1.2, or 1.8 mg per day. In 2014, liraglutide was provided as a 3.0 mg subcutaneous injection daily, as it was approved for the treatment of obesity [59,60]. It is a synthetic molecule, with 97% homology with human GLP-1, produced after adding a 16 carbon fatty-acid side-chains at Lys26 and an Arg34Lys substitution [61].

When liraglutide and placebo were administrated for 26 weeks during a double-blind RCT of 72 PCOS women, the results suggested statistically significant differences regarding weight loss and fertility improvement. The intervention group received 1.8 mg of liraglutide per day subcutaneously. The intervention group presented a mean weight loss of 5.2 kg at the 6-month follow-up in comparison with the placebo group, with a statistically significant difference. As far as fertility improvement is concerned, there were improved menstrual bleeding patterns and a better ovarian morphology (with the ovarian volume significantly decreased by 1.6 mL) in the liraglutide group when compared with the placebo group. In the liraglutide group, there were also significant differences regarding the following findings: SHBG plasma concentrations increased by 7.4 nmol/L and free testosterone serum concentrations decreased by 0.005 nmol/L. In addition, a tendency to statistically—not significantly—reduced AMH concentrations in the liraglutide group was presented at about 8.4 pmol/mL [62].

One more double-blind RCT is presented here, with 72 PCOS, overweight women, who participated in order for researchers to compare the liraglutide treatment of 1.8 mg daily for 26 weeks with placebo therapy. The mean total weight improvement was 5.2 kg in the intervention group in relation to the placebo group. Around half of the participants in the liraglutide group reduced their body weight by over 5%, while, respectively, the percentage of the control participants who achieved this goal was 14%. As far as the reproductive hormonal profile is concerned, the SHBG plasma concentrations increased by 19% and the free testosterone plasma concentrations reduced by 19% in the intervention group when compared with the controls [63].

A 12-week prospective RCT contemplated total body weight reduction regarding hormonal profile changes, when participants were treated with liraglutide, metformin, or roflumilast. Forty-one obese, PCOS women were randomly assigned to three treatment groups. The metformin group commenced with a daily oral dose of 500 mg, later increased to 1000 mg twice per day. Meanwhile, the liraglutide group initiated treatment with a subcutaneous injection of 0.6 mg per day, which was raised to 1.2 mg per day after one week of therapy. Additionally, the roflumilast group received a dosage of 500 mcg. All participants were encouraged to follow a special diet of 500–800 kcal/day. In relation to weight improvement, liraglutide was superior to metformin and better than roflumilast, but without a statistically significant difference. The liraglutide group lost, on average, 3.1 ± 3.5 kg, the roflumilast group 2.1 ± 2.0 kg and the metformin group 0.2 ±1.83 kg. Body mass index decreased by 1.1 ± 1.26 kg/m^2^ in the liraglutide group, 0.8 ± 0.99 kg/m^2^ in the roflumilast group, and 0.1 ± 0.67 kg/m^2^ in the metformin group. No statistically significant differences were found in the hormonal profile of free testosterone, SHBG, LH, and FSH plasma concentrations at the 12-week follow-up, among the therapeutic choices. Menstrual regularity was improved in all treatment groups, with no statistically significant difference [64].

In a prospective open-label RCT of 28 infertile obese women with PCOS, treatment with metformin alone or in combination with a low dose of subcutaneous liraglutide was assessed in relation to weight loss and pregnancy rates after assisted reproductive technology (ART). The intervention lasted 12 weeks. Women in the metformin group (1 g twice per day) lost an average of 7 kg compared with 7.5 kg of weight improvement in the combination group (1 g twice per day of metformin plus 1.2 mg of subcutaneous liraglutide per day), without a statistically significant difference. Regarding pregnancy rates per embryo transfer, there was a statistically significant increase to be mentioned in the combination group, when compared with the metformin-only group, with percentages of 85.7% versus 28.6%, respectively. These beneficial results of the combined therapy on pregnancy rates seemed to stay unalterable during over time, as the outcomes were reevaluated 12 months after the beginning of the study, with the percentages being 69.2% in the combined therapy group and 35.7% in the metformin-only group [65].

A case report was published in 2015, with one obese, infertile PCOS woman who was also diagnosed with DM type 2, who underwent medical therapy with 1.8 mg liraglutide per day for the last two years plus 2 g per day metformin, as she had been diagnosed with DM type 2 at the age of four. She was exposed to liraglutide during the first trimester, as pregnancy was not detected and had a normal pregnancy outcome with a healthy newborn and a successful live birth, while she had also reduced her weight during the last two years by 8 kg [66].

#### 3.1.7. Semaglutide

Semaglutide is another GLP-1 RA regimen; it was approved for use in the USA in 2017, either for adults with DM type 2 or in its higher-dose formulations for long-term weight management in adults with obesity [67]. Its oral formulations are as follows: 3 mg, 7 mg, and 14 mg, once daily. The subcutaneous injection formulations are available in the following dosages: 0.25 mg, 0.5 mg, 1 mg, 1.7 mg, 2 mg, and 2.4 mg, once weekly [68]. Semaglutide is chemically similar to human GLP-1. However, substitutions were made at positions 8 and 34 of GLP-1, where alanine and lysine were replaced by 2-aminoisobutyric acid and arginine, respectively [69]. This medicine increases insulin and stimulates glucagon secretion [70], while it also acts through neuronal pathways to stimulate saturation and energy intake [71]. Studies on how semaglutide affects reproductive health are limited. However, it seems that semaglutide may improve the metabolic parameters of PCOS women, reduce their weight, and improve their insulin sensitivity. It has been surveyed, that semaglutide may have an impact on the hormonal profile by lowering the plasma concentration of free androgens, by improving menstrual regularity and ovarian function, resulting through this way in improved fecundity and higher pregnancy rates [72,73]. Because of the negative research data, which suggest a possibility of harm to the offspring, semaglutide is not currently recommended for pregnant or breastfeeding women. Currently, there is an animal model to be mentioned, which presents an increase in estrogen cycle length and a small reduction in the numbers of corpora lutea [18]. Female rats were included in the animal model and followed three different semaglutide therapy groups: 0.01 mg/kg of semaglutide per day, 0.03 mg/kg/day, or 0.09 mg/kg/day. Overall, in all groups, there was an increase in cycle length and a small decrease in the amount of corpora lutea, which was only presented in doses of semaglutide equal to or greater than 0.03 mg/kg per day. Studies attributed those results of the fertility system to an indirect response of semaglutide on the total body weight change [18].

The total of the above-mentioned references and studies are summarized in Table S1.

## 4. Weight Loss and Male Fertility

The prevalence of male infertility seems to have simultaneously increased with the overall BMI increase in the general world population [33]. As mentioned above, several pathophysiological mechanisms are mediated in order for obesity to result in decreased fecundity. A 2012 systematic review and meta-analysis found that overweight, obese, and morbidly obese men were progressively more likely to present with oligospermia or azoospermia [74]. In relation to published research data, it may be difficult to link increased male BMI to LBRs, due to the confounding female parameters. However, there are elements in the literature that outline reduced fecundity due to male obesity, leading, to a greater extent, to an infertile couple. A prospective cohort study evaluated 114 couples who underwent 172 ART cycles. The authors of this study state that among couples who underwent intracytoplasmic sperm injection, the likelihood of live birth was 84% lower in those with obese male partners than in couples with men with a normal BMI. In the same study, interestingly, the male BMI was unrelated to the positive β-hCG rate, clinical pregnancy rate, or live birth per embryo transfer [75].

Moreover, the practice of recommending male weight reduction in obese infertile couples can be rationalized and further investigated by clinical research, as several pathophysiological mechanisms seem to take part in this complex procedure. Thus, further research in this field may be needed to clarify things further.

Meanwhile, the main points of the current bibliography on the field of male fertility improvement after medical weight loss will be highlighted in the following sections.

### 4.1. Weight Loss by Medical Interventions and Male Fertility

Medical therapies for weight loss and improved fertility are limited, as mentioned before. In this chapter, we provide all data related to each medical therapy used on this field.

#### 4.1.1. Orlistat

As far as orlistat is concerned, there is an interesting, recent animal study, which aimed to assess how orlistat might improve male infertility by interfering with steroidogenesis and steroidogenic genes [75]. Twenty-four adult male rats were randomized equally in groups: normal controls followed a standard rat diet; a high fat diet (HFD) group; a HFD plus preventive orlistat therapy group, which underwent therapy with 10 mg/kg of orlistat twice per day on the same time of HFD for 12 weeks; and lastly, a HFD plus orlistat treatment group, which was directed to follow the same dosage orlistat treatment 7 to 12 weeks after HFD. The HFD plus orlistat-preventive group (HFD+Opr) showed statistically significant decreases in BMI relative to the HFD group, whereas when compared with the HFD+Opr group, concentrations of FSH, LH, testosterone, and estradiol were significantly lower in the HFD plus orlistat-therapy group. Orlistat treatment showed statistically significant improvements in both orlistat therapy groups, when compared with the HFD group, regarding sperm count, motility, and sperm rapid forward movement. The results clarified that orlistat may reveal the negative effects of HFD-induced obesity by up-regulating steroidogenesis-related genes, especially when it is provided as a preventive medical intervention. Because of these results, there is a new tendency that orlistat might be a new therapeutic option for obesity-induced male subfertility, regardless of its effect on body weight management [76].

Another animal study examined the effects of orlistat on the fertility of male rats, who also followed an HFD. Eighteen adult male rats were grouped equally in three populations: normal controls, the HFD group, and the HFD plus orlistat group, who followed medical therapy of 10 mg/kg of orlistat per day for 12 weeks simultaneously with a HFD (HFD+O group). The results demonstrated that orlistat improved male fertility potential in obese mice by targeting lactate metabolism in the testis, so because of this data, orlistat may have promising positive results regarding the fertility potential of obese men [77].

#### 4.1.2. Metformin

Metformin has been studied in relation to male fertility, as far as ED, steroidogenesis, and spermatogenesis are concerned [78].

Firstly, a human study examined the impact of decreasing BMI after metformin therapy on hormonal profile and seminal fluid analysis in obese infertile males [79]. Eighteen obese patients with a BMI of 30–40 kg/m^2^ and mean ages of 22 to 42 years, suffering from idiopathic asthenozoospermia, were included in the study. They were evaluated for standard semen analysis according to WHO and for reproductive hormonal profiles such as FSH, LH, testosterone and estradiol plasma concentrations at baseline and at 12 weeks follow-up. The metformin group was treated with 850 mg twice daily, orally for 12 weeks and was compared with the controls, treated with the same dosage of metformin and normal BMI. The results demonstrated a statistically significant decrease in sperm count and activity after 12 weeks of treatment with metformin. The mean BMI decreased significantly (from 35.93 ± 5.7 kg/m^2^ to 34.85 ± 5.2 kg/m^2^), while there were not statistically significant differences in the hormonal plasma profile between baseline and 3-month follow-up, between the groups [79]. A prospective RCT included 30 male patients with ED and IR, where ED was evaluated based on the International Index of Erectile Function 5 (IIEF-5) and IR based on homeostasis model assessment (HOMA) with a HOMA score over 3 [80]. Patients were divided into two groups: 850 mg of metformin therapy at lunch and 850 mg at dinner and a placebo group, all of which were all evaluated for 4 months. The results show that metformin treatment resulted in a significant increase in the IIEF-5 score and a significant decrease in the HOMA score, both examined at the 2-month follow-up and 4-month follow-up. Body mass index was decreased in the metformin group in months 2 and 4 compared with their baseline numbers, whereas there were no changes to be mentioned in the BMI of the placebo group at 2 and 4 months, respectively [80].

An animal study on 5-to-6-week-old male mice investigated how a 6-week metformin therapy (28 mg/kg per day) influenced the male fertility of obese mice after they were fed with a 16-week HFD, in comparison with the controls, who followed a control diet (CD) [81]. This animal model suggests that metformin treatment of HFD male mice improved glucose tolerance at a level of 12%, without presenting a change in body weight or adiposity. This therapy also restored testicular morphology, increased significantly sperm motility and sperm number, which could be bound to MII oocytes, and, importantly, reduced sperm intracellular reactive oxygen species (ROS) concentrations and oxidative DNA lesions to levels respective of the CD group. As far as embryos are concerned, the metformin therapy father-group was finally linked to better fetal body weight and length. Through these observations, there is a new option of following a short-term metformin treatment in obese, infertile men in order to improve their subfertility, without the need for a specific reduction in body weight/adiposity [81]. Similarly, another animal model about metformin’s antioxidant effects on the male reproductive system included seven obese male mice, that were treated with an HFD and then metformin in water for 8 weeks. Their results suggested that metformin improves obese male fertility by alleviating injury of the blood–testis–barrier structure and permeability, as it restores the disordered related proteins and by improving the oxidative stress in Sertoli cells [82].

#### 4.1.3. Exenatide

In a mice-cell model of therapy of dapagliflozin and exenatide in several dosages (especially of sub-pharmacologic, pharmacologic, and supra-pharmacologic concentrations of dapagliflozin 50, 500, and 5000 nM, respectively, and/or exenatide of 2.5, 25, and 250 pM, respectively),researchers aimed to analyze the effects of this medical treatment on Sertoli cells’ metabolism. The medical treatment of exenatide plus dapagliflozin seemed to improve sperm production and quality by reducing lactate production by Sertoli cells [83]. Another animal model with mice aimed to interpret the effect of exenatide on sperm quality and inflammation within the testis. After 12 weeks of either CD or HFD on a total of 21 mice, grouped by half between the groups, they followed the medical intervention of either saline or intraperitoneal exenatide (24 nmol/kg daily) for 8 more weeks. The exenatide group showed a statistically significant decrease in total body weight compared with the control group. Regarding hormone profile alterations, the serum testosterone concentrations were significantly decreased in the HFD group when compared with the CD group, without an important difference related to exenatide treatment. The sperm quality and the inflammatory profile of the testis were evaluated and their results concluded the following: the exenatide intervention group presented significantly improved sperm motility and activity and significantly reduced inflammatory cytokines, such as levels of tumor necrosis factor (TNF-a), within the testis, and, importantly, the serum testosterone levels were decreased when compared with the controls [84].

#### 4.1.4. Liraglutide

Recent studies suggest that the use of GLP-1 RAs in obese males may enhance sperm metabolism and sperm motility, and result in positive effects on the human Sertoli cells. Weight improvement associated with GLP-1 RAs is correlated with amelioration in sperm count, concentration, and motility [85]. Regarding liraglutide, there are two recent RCTs to be presented. A 4-month, prospective RCT included 110 men of childbearing age with metabolic hypogonadism. Participants were divided into three groups: patients treated with gonadotropins, patients on liraglutide of 3 mg once per day and patients on transdermal testosterone of 60 mg per day. The liraglutide group presented the best outcomes when compared with the baseline measurements and the other groups: a significant reduction in total body weight of 10.3% (116 ± 10 vs. 104 ± 6 kg), a BMI reduced by 16.7% (36 ± 3 vs. 30 ± 2 kg/m^2^), a significantly increased serum total testosterone and SHBG concentrations. Interestingly, plasma concentrations of FSH and LH, as well as all sperm parameters, including sperm motility and IIEF-5 score, showed a significant increase as well [86]. In another 16-week pilot study of 30 middle-aged obese men with functional hypogonadism, liraglutide presented also statistically significant differences in comparison with testosterone replacement treatment. The participants were randomly divided into two groups: half of them started on treatment of 3.0 mg liraglutide subcutaneously and half of them on treatment of 50 mg of 1% transdermal gel of testosterone replacement therapy. In the liraglutide group, the SHBG, LH and FSH plasma concentrations tended to increase significantly. Participants treated with liraglutide lost a mean total body weight of 6.0 ± 3.2% compared with 0.8±3.3% of the total body weight reduction in the testosterone group [87]. A case report is mentioned about this special medicine, where a 35-year-old man with primary and idiopathic infertility for one year started to use liraglutide of 0.6 mg daily, for 2 months and lost 2 kg during this period. The first new spermiogram showed normal semen volume, normal leucocyte concentration and sperm concentration of 0.01 × 10^6^ sperm/mL, with no sperm motility. Four months after liraglutide interruption, a new spermiogram was presented, this time with normal sperm volume, normal leucocyte concentration, 8.7 × 10^6^ sperm/mL concentration, normal sperm motility, and 2.5% normal sperm morphology. Five months after medical interruption, the semen analysis was normal for all of the parameters evaluated. After this time period, the oocyte transfer was held and resulted in a successful twin-pregnancy and live birth at a pregnancy age of 36 weeks [88].

All the above-mentioned references and studies are summarized in Table S2.

## 5. Discussion

Overall, in this review, all possible effects of medically induced weight improvement on the fertility of women and men were assessed, after a literature search of human and animal studies. These effects of medically induced weight loss on female fecundity were studied regarding the following fertility outcomes: reproductive hormonal profile, ovulation rates, TTP, implantation rates, pregnancy rates, normal embryo development, and LBRs. Regarding male fertility, the outcomes outlined were as follows: the reproductive hormonal profile, sperm motility, morphology and movement, the weight of other male reproductive organs, and sexual function. Primarily, this review aims to shape a precise opinion about whether medical weight loss provides amelioration of fertility or not and in relation to which fertility outcomes. Furthermore, which medical therapy is the most effective and what quantitative percentage of weight should be lost during a period of time. Finally, whether the fecundity improvement is a direct outcome of the medicine action or an indirect effect of the body weight change.

Infertility improvement due to weight loss by medical therapy remains a controversial issue, without relatively many human studies, especially regarding male fertility. On the contrary, there are a few published animal models that present the results of medically induced weight loss effects on fertility, while a large percentage of the existing published human studies are on the special population of obese, infertile PCOS women. As far as the newer GLP-1 RAs are concerned, data are still emerging from some animal studies or limited existing human RCTs. In relation to male fertility improvement after medical weight loss, most published studies are animal models, while there is only limited known data on humans about metformin and liraglutide results.

More specifically, orlistat seemed to provide statistically significant weight reduction and amelioration of the female hormonal profile [41,42,43,44]. In addition, when orlistat was compared to metformin, it presented better ovulation and conception rates [43,44]. Regarding the male reproduction system, orlistat provided important weight improvement and amelioration of the reproductive hormonal profile, sperm count, and sperm motility [76]. Metformin demonstrated better results as a combined therapy with other medicines, such as saxagliptin or exenatide [46,52]. As a combined therapy, metformin provided a statistically significant weight improvement and significant differences in female reproductive hormones, such as free testosterone plasma concentrations, SHBG concentrations, and better menstrual cyclicity. Exenatide provided statistically significant weight change and fertility improvement regarding reproductive hormonal profile, menstrual cyclicity, and spontaneous pregnancy rates. In most published human studies or animal models, exenatide has been compared with other medicines, such as metformin [53,54]. Moreover, metformin and exenatide evidenced positive results on the male reproductive system after weight improvement, resulting in statistically significant changes in the male reproductive hormonal profile, sperm motility, morphology, and count [81,84]. Dulaglutide seemed to provide statistically significant weight change and fertility improvement, in relation to the female reproductive hormonal profile and ovarian morphology, according to a published animal model [58]. However, it is not certain whether this amelioration was performed by a direct effect of dulaglutide on the female fertility system or by the weight change and its indirect results on the female reproductive system. There have been some human studies about liraglutide, which did not all show a significant improvement in fertility outcomes [62,63,65]. However, we should wait for more studies to come out in the field. Newer medical choices may provide significant weight loss and female fertility amelioration, regarding menstrual cyclicity and ovarian morphology [18]. However, the total body weight change remains the most important influencing factor.

Overall, according to already published data in human studies, there is fertility improvement after medically induced weight loss, indeed. The major critical factor of this amelioration seems to be the total amount of body weight change. The precise quantitative percentage needed seems to be a weight reduction of 5% of the initial total body weight (loss of 5–6 kg), which seems to be successful, regarding fecundity improvement [40,44,52,53,54,62,65]. This weight change goal should be achieved within a period of time before succeeding in a clinical pregnancy. The current studies suggest that the longer this period is (16 to 26 weeks), the better results will be presented regarding the total of medically induced weight loss and the fertility outcomes [41,52,53,54,62]. The fertility outcomes upon which medically induced weight loss provides positive results are the following: female reproductive hormonal profile, menstrual cyclicity and regularity, ovulation/conception rates, and pregnancy rates [41,42,43,44,52,53,54,62,63,65]. Regarding the male reproductive system, significant results have been presented after medically induced weight improvement in relation to male hormonal profile, sperm motility, movement and morphology, weight of male reproductive organs and sexual function [76,80,81,84,86,87]. The newer injectable GLP-1 RAs (exenatide and liraglutide) seem to achieve more weight loss from the initial body weight compared with older therapeutic choices, such as metformin and orlistat, in comparative human studies; this was accompanied with a more favorable improvement in female fertility, according to reproductive hormonal profile, menstrual cyclicity, and spontaneous pregnancy rates [52,53,54,62,63,65]. GLP-1 analogues have shown promising effects on fecundity by improving ovulation rates and regulating the menstrual cycle [89]. However, it is still the early days for these new medicine drugs, due to the limited number of published human studies. The total fertility improvement of women and men after medical weight loss seems to result from the indirect effects that total body weight change provides reproductive systems.

Lastly, there are data that present no statistically significant results upon fertility improvement after medically induced weight loss. An RCT presents no significant differences of pregnancy rates, conception rates or LBRs when orlistat was compared with placebo [45]. Another study about liraglutide resulted in no significant differences in female reproductive hormonal profile and menstrual regularity [62]. A prospective study about metformin presented no significant differences in female reproductive hormonal profile [46]. These studies are only a few and were carried out in a short intervention period of time (12 to 16 weeks), without following a mandatory lifestyle modification of diet and without resulting, at least, in a modest weight loss of 5% of the total initial body weight [45,46,64].

All things considered, modest weight loss after medication and the duration of the treatment are important factors for fertility improvement. The newer promising GLP-1 RAs used in weight management show expectations regarding fertility improvement (before fertility management), but more studies are needed to confirm this. Future research should target on providing answers about whether medical weight loss therapies affect fertility indirectly through weight loss or by a possible direct action on the reproductive system.

## Data Availability

All data analysed in this study are available within the paper and its Supplementary Information.

## Supplementary Materials

The following supporting information can be downloaded at: https://www.mdpi.com/article/10.3390/ijms25031909/s1, References [18,41,42,43,44,45,47,52,53,54,58,62,63,64,65,66,76,77,78,79,80,81,84,86,87,88] are citied in the Supplemental Materials.
