# Supplementary material for: Infertility Improvement after Medical Weight Loss in Women and Men: A Review of the Literature
Source: Int J Mol Sci. 2024 Feb 5;25(3):1909. doi: 10.3390/ijms25031909 (PMC10856238; doi:10.3390/ijms25031909)
Supplement: Supplementary file 1 [file ijms-25-01909-s001.zip › ijms-2814591-supplementary.pdf]

Table S1: All published data related to female fertility system outcomes after medical-induced weight loss.

Reference	Type of study and duration	Population	Groups	Weight change	Fertility outcomes
[41]	prospective study- 6 months	120 women with body mass index (BMI) over 25kg/m^2	group I (N=60) on orlistat 120 mg twice daily, group II (N=60) on lifestyle modification program	statistically significant difference at weight pre- and post- treatment in both groups	statistically significant increase of post-treatment luteinizing hormone (LH), significant reduce of pre- and post-treatment free testosterone levels and anti-Müllerian hormone (AMH) in the olristat group and significant difference regarding pregnancy rates
[42]	prospective cohort study- 24- week	81 obese women	(N= 61) obese polycystic ovarian syndrome (PCOS) women, N= 20 overweight and obese controls, on energy-restricted diet, physical exercise plus orlistat administration	statistically significant reduced BMI in both PCOS and control women, especially during the first trimester(from 34.83+-6.39 to 31.90+-6.09kg/m^2)	LH and sex hormone-binding globulin (SHBG) significantly increased after 12 weeks, testosterone concentrations statistically significantly reduced
[43]	prospective cohort study- 24-week	32 obese women	(N=18) PCOS women,14 obese controls, with normal menstrual cycles on energy-restricted diet plus orlistat	statistically significant weight improvement (BMI in the PCOS group from 36.00+-1.29kg/m^2 to 30.36+-1.18kg/m^2)	testosterone concentrations statistically significantly reduced in the PCOS group during the first trimester (from 83.26 +-6.86ng/dl to 61.50+-4.97ng/dL)
[44]	Randomized control trial (RCT)-3-month	90 PCOS women, with BMI >23kg/m^2	orlistat 120mg x2 (N=30), metformin 500mg x3 (N=30) in combination with lifestyle interventions, compared with (N=30)	both medical treatments: similar results related to weight improvement and BMI (7.81±0.66kg reduction in the orlistat group, 7.78±0.57kg in the metformin group)	ovulation rate: 33.3%, 23.35% with orlistat and metformin group respectively, conception rates 40%, 16.7% and 3.3% in the orlistat group, the metformin group and the control group, respectively

			controls, on lifestyle modifications only		
[45]	RCT - 12 week	877 obese women, with BMI>over 25kg/m^2	on orlistat (n = 439) or placebo (n = 438)	weight loss −2.49 kg in the orlistat group, −1.22 kg in the placebo group, statistically significant differently	live birth rates (LBRs): not significantly different between the groups, no significant differences in relation to the pregnancy rates or clinical pregnancy
[47]	prospective pilot study - 16-week	34 PCOS, prediabetes women	metformin 2000mg once /day group (N=12), saxagliptin 5mg once/day group (N=11) and combination group (N=11)	BMI reduced significantly in saxagliptin and combination group (pre and post-therapy respectively: from 37.2+-6.8 to 36.7+-7.4kg/m^2 and from 43.8+-10.5 to 42+-10.2kg/m^2)	menstrual cyclicity better in the combined therapy group, dehydroepiandrosterone-sulfate (DHEAS), testosterone and FAI: significantly lower and SHBG levels were increased, but not with a significant difference
[52]	open-label prospective, randomized study-24- week	60 overweight/obese PCOS women	metformin group:1000mg twice daily, exenatide group: 10 µg twice daily and combined group	statistically significant weight loss in all three groups, better in the combination group(mean weight loss of 6 +-0.5 kg for combined group, 3.2+-0.1kg for exenatide and 1.6+-0.2kg for metformin)	total testosterone concentrations, FAI: significantly decreased in all groups, SHBG concentrations improved, but without significant difference, menstrual cyclicity got improved statistically significantly in the combined treatment group
[53]	open-label RCT-24-week	176 overweight/obese PCOS women	metformin group (N=88): 1000mg twice per day or exenatide group (N=88): 10 µg twice per day for the initial 12 weeks, following 12 other weeks,with metformin-only-therapy	exenatide: statistically significant weight loss vs metformin: - 4.29 ± 1.29 kg versus -2.28 ± 0.55, respectively, and total fat reduction of 4.67 ± 0.09% versus 1.11±0.32%, respectively	menstrual cyclicity: improved in the exenatide group, with higher rate of spontaneous pregnancy
[54]	RCT- 24-week	160 PCOS women, BMI over 24kg/m^2	exenatide group (N=80): 5 µg twice daily, increased to 10 µg twice	statistically significant weight loss and BMI improvement between the	total pregnancy rates were 79.2% in the exenatide group and 76% in the metformin group without

			daily after 4 weeks, for 12 weeks, metformin group (N=80): 500 mg twice per day, gradually multiplied up to 1000 mg twice daily, for 12 weeks. The following 12 weeks, all treated with metformin-only- therapy	groups, with exenatide presenting better results (−5.21+-3.94kg in the exenatide group, −3.55 +-2.13kg weight reduction in the metformin group, and respectively, −2.16+-1.53kg/m^2 and −1.39+-0.89kg/m^2)	significant difference
[58]	animal model - 6 weeks	50 female rats	medical-induced-PCOS group (n=40) and controls (n=10). The medical induced-PCOS group further divided into :only PCOS (n=10), PCOS +50 μg/Kg of dulaglutide (n=10), PCOS +150 μg/Kg of dulaglutide (n=10), PCOS +450 μg/Kg of dulaglutide (n=10)	statistically significantly reduced body weight in a dose-depending way	androgen concentrations of the dulaglutide group significantly decreased, SHBG significantly increased and better ovarian function and morphology
[62]	RCT - 26 week	72 PCOS women	intervention group (N=48) on 1.8mg of liraglutide per day subcutaneously, and controls on placebo (N=24)	intervention group presented at 6-month follow-up: statistically significant different mean weight loss of 5.2 kg compared with control group	liraglutide group: improved menstrual bleeding pattern, better ovarian volume significantly decreased by 1.6ml in relation to placebo, SHBG increased by 7.4 nmol/L, free testosterone decreased by 0.005 nmol/L, significantly, a tendency to not-significantly reduced AMH at about 8.4pmol/ml

[63]	double blind RCT - 26 weeks	72 women, with PCOS and BMI over 25kg/m^2	liraglutide treatment of 1.8mg daily (N=48) compared with placebo therapy (N=24)	mean total weight improvement:5.2kg in the intervention group in relation to placebo. 55% of the participants in the liraglutide group reduced their body weight over 5% and 14% of the controls respectively	SHBG: increased by 19% and free testosterone levels reduced by 19% in the intervention group
[64]	RCT - 12- week	41 obese PCOS women	metformin group started on 500mg orally, daily upon 1000mg twice per day (N=13), liraglutide group on a subcutaneous injection of 0.6mg per day increased to 1.2mg per day after one week of therapy (N=14) and 500mcg of roflumilast (N=14) plus all followed diet 500-800kcal/day	liraglutide group lost on average 3.1 ± 3.5 kg, roflumilast group 2.1 ± 2.0 kg and metformin group 0.2 ±1.83 kg, respectively. BMI decreased by 1.1 ± 1.26 kg/m^2 by liraglutide, 0.8 ± 0.99 kg/m^2 by roflumilast, 0.1 ± 0.67 kg/m2 by metformin	no statistically significant differences on free testosterone, SHBG, LH and FSH, on 12-week follow-up, among three groups. Menstrual regularity got improved in all groups with no statistically significant difference
[65]	prospective RCT - 12 weeks	28 infertile obese PCOS women on assisted reproductive technology (ARTs) progress	metformin group (1g twice per day, N=14) and combined group (1g twice per day of metformin plus 1.2mg of subcutaneous liraglutide per day, N=14)	metformin group lost an average of 7 kg compared to 7.5 kg of weight improvement in the combination group; no statistically significant difference	pregnancy rates per embryo transfer: statistically significantly increased in the combined therapy group compared to the metformin- group (85.7% versus 28.6%, respectively). 12 weeks after: 69.2% in the combined therapy group and 35.7% in the metformin only
[66]	case report – 2 years	1 woman with diabetes mellitus (DM) type 2	liraglutide 1.8mg per day for the last two years plus 2 gr per day metformin	reduced her weight by 8kg	pregnancy with a normal pregnancy outcome with a healthy newborn and a successful live birth

[18]	animal study – 4 weeks	female rats	female and male rats; males were on observation for 4 weeks prior to mating, females for 2 weeks prior to mating and till 17th gestation day, divided into 3 groups (0.01, 0.03 and 0.09 mg/kg/day of subcutaneous semaglutide)	statistically significant reduction in total body weight	increased menstrual cycle length in all groups; a small decrease in numbers of corpora lutea, only in doses of equal or greater than 0.03mg/kg/day.

Table S2: All published data related to male fertility system outcomes after medical-induced weight loss.

Reference	Type of study and duration	Population	Groups	Weight change	Fertility outcomes
[76]	animal study- 12 weeks	24 adult male rats	controls on control diet (CD) (N=6), high fat diet (HFD) group (N=6), HFD plus preventive orlistat therapy group for 12 weeks (N=6) and HFD plus orlistat treatment group (10mg/kg/day), 7 to 12 weeks after HFD	HFD plus orlistat-preventive group showed statistically significant decreased BMI relative to the HFD group	HFD+orlistat therapy group showed statistically significantly reduced concentrations of FSH, LH, testosterone and estradiol when compared to the orlistat preventive group, both orlistat treatments: significant improvement on sperm count, motility and rapid forward movement, when compared to HFD group

			(N=6)		
[77]	animal study - 12 weeks	18 adult male rats	controls on CD (N=6), HFD group (N=6), HFD plus orlistat therapy group (10mg/kg/day) for 12 weeks (N=6)	not clearly clarified	orlistat improves male fertility by targeting lactate metabolism in testis
[79]	prospective cohort study - 12 weeks	18 obese males with BMI 30-40 kg/m^2 , mean ages of 22 to 42 years, under idiopathic asthenozoospermia	metformin group on 850 mg twice daily, orally (N=9) and controls (N=9), treated with the same dosage of metformin but normal BMI	mean BMI was decreased statistically significantly (from 35.93 ± 5.7 to 34.85 ± 5.2kg/m2) in metformin group	statistically significant decrease in sperm count and activity after 12 weeks of metformin; no significant differences on serum concentrations of LH, FSH, estradiol, testosterone between baseline and 3 month-follow up, in the metformin group
[80]	prospective RCT- 4-month	30 male patients with ED and IR	metformin group therapy of 1700mg per day (N= 17) or placebo group therapy (N= 13)	no changes to be mentioned in BMI of placebo-group in 2 and 4 months, respectively	significant increased International Index of Erectile Function 5 (IIEF-5) score in the metformin group
[81]	animal model - 22 week	25 male rats	controls on CD (N=9), HFD group for 22 weeks (N=8), HFD+metformin of 28mg/kg/day group for 6 weeks after 16-week HFD first period (N=8)	metformin treatment of HFD male mice improved 12% glucose tolerance, without statistically significant change in body weight or adiposity	metformin therapy restored testicular morphology, increased significantly sperm motility and number; metformin therapy group: better fetal body weight and length
[84]	animal model- 20 weeks	21 mice	controls on CD (N=5), HFD plus saline (N=8), HFD plus exenatide of 24 nmol/kg/day, intraperitoneal (N=8)	statistically significant decreased total body weight in exenatide group	serum testosterone concentrations statistically significantly decreased in the HFD group when compared to the CD group, without an important difference related to the exenatide treatment; exenatide intervention group: significantly improved sperm motility and activity
[86]	prospective RCT- 4	110 men of childbearing age	group A (*n* = 35):	a significant reduction in total	In the liraglutide group: significantly increased

	month	with metabolic hypogonadism	urofollitropin 150 IU subcutaneously, three times a week. Group B (*n* = 35): liraglutide 0.6 mg subcutaneously every day for the first week and on continuous augmentation. Group C (*n* = 40): transdermal testosterone gel (2%) at 60 mg every day	body weight of 10.3% in the liraglutide group (116 ± 10 vs. 104 ± 6 Kg,) and a BMI reduced by 16.7% in this group (36 ± 3 vs. 30 ± 2 Kg/m^2)	serum total testosterone and SHBG concentrations; FSH and LH plasma concentrations, sperm motility and IIEF-5 score: significantly increased
[87]	pilot study – 16 weeks	30 middle-aged obese men with functional hypogonadism	group of liraglutide 3.0 mg subcutaneously (n=15) and group of 50 mg of 1% transdermal gel of testosterone replacement treatment (n=15)	liraglutide group lost a mean total body weight of 6.0 ± 3.2% compared to a 0.8 ± 3.3% in the testosterone group	in the liraglutide group: SHBG tended to increase, significant increase in LH and FSH plasma concentrations.
[88]	case report - 2 month	one 35-year-old man with primary, idiopathic infertility since 1 year	liraglutide of 0.6mg daily, for 2 months	-2kg during this period	first spermiogram: normal semen volume, leucocyte concentration and sperm concentration of 0.01 × 10^6 sperm/ml, with no sperm motility; 4 months after liraglutide interruption, spermiogram with normal sperm volume, leucocyte concentration, 8.7 × 10^6 sperm/ml concentration, normal motility and 2.5% normal morphology; 5 months after medical interruption, semen analysis normal for all parameters; when oocyte transfer was held, resulted in successful full term pregnancy and live birth
